# Effects of stress on school bullying behavior among secondary school students: Moderating effects of gender and grade level

**DOI:** 10.3389/fpsyg.2023.1074476

**Published:** 2023-03-15

**Authors:** Siliang Luo, Yongfei Ban, Tianlong Qiu, Changyou Liu

**Affiliations:** School of Educational Sciences, Anshun University, Anshun, Guizhou, China

**Keywords:** secondary school students, stress, school bullying behavior, gender, grade level

## Abstract

This study investigated the effects of stress on school bullying behaviors among middle school students, and the moderating role of gender and grade level in this relationship. To this end, the Olweus Child Bullying Questionnaire (OBVQ) secondary school version of the child bullying questionnaire and the stressor scale for secondary school students were used to survey 3,566 secondary school students in Guizhou Province, and the data were statistically analyzed. Results showed that stress was significantly and positively associated with school bullying among secondary school students. Furthermore, both gender and grade moderated the relationship between stress and school bullying, showing that boys and middle school children are more likely to engage in bullying than girls and high school students, respectively. The results of the study provide a theoretical basis for the prevention and intervention of school bullying behaviors among middle school students.

## 1. Introduction

School bullying has been defined as intentionally aggressive behavior that occurs repeatedly between victims and perpetrators due to a power imbalance (Olweus, 2013). Bullying in schools is a global youth problem that negatively affects the health and educational outcomes of young people (UNESCO, 2017; Armitage, 2021). UNESCO stated that nearly one-third of students report that they have experienced bullying in school in the past month (UNESCO, 2019). School bullying can have serious physical and psychological effects on adolescents if it is not addressed in a timely and effective manner. For bullies themselves, it usually leads to emotional and behavioral problems, such as anxiety (Eyuboglu et al., 2021; Luo et al., 2022), depression (Eyuboglu et al., 2021), insomnia (Fábia Carvalho et al., 2020), poor academic performance (Riffle et al., 2021), violent criminal behavior (Baldry, 2014), self-harm (Eyuboglu et al., 2021; Myklestad and Straiton, 2021; Luo et al., 2022), suicidal ideation (Husky et al., 2022), and suicidal behavior (Benatov et al., 2022).

Adolescents often experience higher levels of stress, which is more likely to lead to maladaptive stress reactions (Stelzig and Sevecke, 2019). Stress is “the subjective assessment of an individual’s response to various stimuli in life” (Liu and Zhang, 2003), and it is one of the important environmental indicators that influence individuals’ psychological and behavioral development (Koss and Gunnar, 2017; Engel and Gunnar, 2020). The theoretical model of psychological stress states that stress can alter the steady state within the organism and cause significant psychological and behavioral harm to individuals if it is too strong or lasts for too long (Schroeder et al., 2018). Studies have been conducted to reveal the mechanisms by which stress triggers or exacerbates aggressive behavior (Mendonça et al., 2019; Farrell et al., 2020; Felippe et al., 2021). Previous studies found a strong relationship between stress and aggressive behavior (Demichelis et al., 2022; Hasegawa et al., 2022). Some of them confirmed that early stress (e.g., childhood adversity, early parenting styles) can lead to increased aggression later in life (Connell et al., 2015; Bernhard et al., 2018). In addition, chronic stress over time may lead to excessive adaptive changes, which may result in maladaptive aggression in adulthood (Tielbeek et al., 2018). In recent years, research on adolescents has found that impulsive aggression may be associated with higher levels of environmental stress (Liu et al., 2018; Liu and Liu, 2021), Individuals with PTSD are more likely to engage in physical aggression (Saunderson et al., 2022). Zhang et al. (2021) noted that the higher the pressure adolescents receive from their parents, the greater the risk of perpetrating bullying or being bullied. Therefore, this study hypothesizes that stress can significantly predict larger effects of school bullying behavior.

Mcgue and Jr (1984) noted that for most psychological and physiological variables, age and gender have a strong influence. Numerous studies have shown age- and gender-related differences in the experience and management of stress (Schroeder et al., 2018; Stelzig and Sevecke, 2019). Schroeder et al. (2018) concluded that men tend to be more vulnerable to the long-term effects of early life and adolescent stress. Numerous studies on school bullying have also revealed gender and grade (age) differences in school bullying. In terms of gender differences in bullying, the number of boys who bully is higher than the number of girls (Olweus, 1993; Eyuboglu et al., 2021), especially at the secondary level (Rivers and Smith, 1994); there are also significant differences in both the form and the type of bullying (Olweus, 1994; Smith and Sharp, 1994), with boys using direct bullying more often and girls favoring indirect bullying. In terms of grade (age) differences, the incidence of school bullying behavior was shown to decrease as grade (age) increased (Boulton and Undeeerwood, 1992; Whitney and Smith, 1993). Moreover, some studies revealed that age and gender act as moderating factors between adolescent exposure to violence and emotional/behavioral problems (Bordin et al., 2022). Therefore, this study hypothesizes that grade and gender moderated the relationship between stress and school bullying.

Previous studies have focused more on the influence of distal environmental triggers (early stress) on aggression, and on the change of school bullying behavior from elementary school to middle school level. Our study focuses more on the influence of proximal environmental triggers (recent triggers), thus expanding the influence of stress on aggression in this study. It also focuses on the change of school bullying behavior from middle school to high school, which complements previous studies.

**Figure 1:**
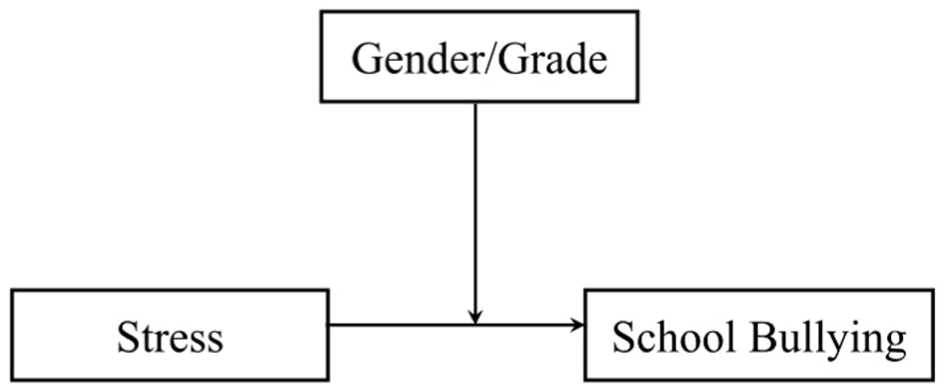
Model diagram of the role of gender and grade level in the relationship between stress and school bullying behavior.

This study hypothesizes that stress has significantly impact on school bullying behavior among secondary school students, while gender and grade level play a moderating role between the two. Figure 1 presents the research model.

## 2. Materials and methods

### 2.1. Study object

This study adopted a simple random sampling method and selected a total of 3,600 students from middle schools in the Anshun, Liupanshui, Bijie, and Qiannan areas of Guizhou Province from May to June 2019. This study was cross-sectional design. According to the Chinese education system, secondary school includes the seventh grade, the eighth grade, the ninth grade, the tenth grade, the eleventh grade, and the twelfth grade. The data of students who did not answer all items or chose the same answer for all items were excluded. Of these, 3,566 responded with valid answers to the questionnaires—a 99% return rate. There were 1,539 male students (43.2%) and 2,027 female students (56.8%); 436 students (12.2%) in the seventh grade, 647 students (18.1%) in the eighth grade, 582 students (16.3%) in the ninth grade, 562 students (15.8%) in the tenth grade, 1,108 students (31.1%) in the eleventh grade, and 231 (6.5%) in the twelfth grade (see Table 1). Informed consent was obtained from all respondents. The studies involving human participants were reviewed and approved by School of Educational Sciences, Anshun University, China.

**Table 1:** Demographic information (*n* = 3,566).

Variables		*n*	%	Variables		*n*	%
Gender	Male	1,539	43.2	Grade	Grade 7	436	12.2
	Female	2027	56.8		Grade 8	647	18.1
					Grade 9	582	16.3
					Grade 10	562	15.8
					Grade 11	1,108	31.1
					Grade 12	231	6.5

### 2.2. Research tools

#### 2.2.1. Child bullying questionnaire OBVQ secondary school edition

In this study, the Olweus Child Bullying Questionnaire (OBVQ) Secondary School Version, revised by Zhang et al. (1999), was used to measure school bullying. The question, “(1) Have you bullied other students in any way at school this semester?” was used to identify child bullies. The type of bullying was measured by a subscale (six items), which was selected to examine the frequency of student bullying during that semester. It is divided into three parts: verbal bullying, relationship bullying and physical bullying. Scoring is on a four-point scale Score, rated from 0 to 4 (0 = *not occurring*, 1 = *only once or twice*, 2 = *two or three times a month*, 3 = *once a week*, and 4 = *several times a week*). The Cronbach’s ɑ coefficient for this subscale was 0.81, and the retest reliability was 0.77; Cronbach’s ɑ for this study was 0.869.

#### 2.2.2. Stressor scale for secondary school students

This study used the stressor scale for secondary school students developed by Zheng and Chen (1999), which consists of 39 stressful events that can cause stress reactions in secondary school students, including seven factors: academic learning stress, teacher stress, family environment stress, parenting style stress, peer friend stress, social and cultural stress, and body stress. High scores on the scale indicate more stressors, while low scores indicate the opposite. The Cronbach’s ɑ coefficient for this scale was 0.93 and the retest reliability was 0.86. Cronbach’s ɑ for this study was 0.928.

### 2.3. Statistical methods

This study was conducted using a group questionnaire with standardized administration procedures. SPSS 24.0 and the macro program Process developed by Hayes were used for data analysis and processing. The moderating effect of gender and grade level on the stress and school bullying behavior was explored using the Bootstrap method proposed by Hayes (2013), with a sample size of 5,000 and Bootstrap CI using bias correction at 95% confidence level. Model testing criteria were used for confidence intervals not containing 0 and *p* < 0.05 and the model established met the fit criteria (Hayes, 2013). The direct and interactive effects of stress, gender, and grade on school bullying were then analyzed, as well as the specific moderating effects of gender and grade on said relationship.

## 3. Results

### 3.1. Descriptive statistics and correlation analysis of the main study variables

Pearson correlation analysis found statistically significant correlations between gender, grade, stress, and school bullying (*r* = |0.19–0.28|, *p* < 0.01), and no statistically significant correlations between grade, gender, and stress (*r*_gender_ = −0.03, *r*_grade_ = −0.01, *p* > 0.05). According to the MacArthur method determination criteria, M was determined to be a moderating variable if it was not significantly correlated with *X* and *Y* or if the correlation was small (Kraemer et al., 2008). Based on the prior hypothesis and the related results, one concludes that gender and grade can be used as moderating variables. The descriptive statistics are shown in Table 2.

**Table 2:** Descriptive statistics and correlation analysis of each variable (*r*, *n* = 3,566).

	*M* ± SD	Gender	Grade	Stress	Bullying
Gender	1.57 ± 0.50	1			
Grade	1.53 ± 0.50	0.12^**^	1		
Stress	0.88 ± 0.53	−0.03	−0.01	1	
Bullying	0.16 ± 0.44	−0.19**	−0.0.19^**^	0.28^**^	1

^*^p < 0.05, ^**^p < 0.01, ^***^p < 0.001.

### 3.2. Effects of gender and grade level on the relationship between stress and school bullying among secondary school students

The variables were transformed prior to effect analysis, and the continuous variables “school bullying” and “stress” were transformed in a standardized manner. The categorical variables “gender” and “grade” were coded as male = 1, female = 0, middle school = 1, and high school = 0. The analysis showed that the confidence intervals of the coefficients of the established model did not contain zero, all of them were statistically significant, as were the interaction effects, and the model met the fit criteria.

#### 3.2.1. The moderating role of gender in stress and school bullying behavior

Bootstrap analysis found that stress and gender significantly predicted school bullying, explaining 12.6% of the total variance in school bullying [*F*(3, 3,562) = 171.131, *p* < 0.001]. The interaction between the first two explained 1.5% of the total variance [*F*(1, 3,562) = 58.98, *p* < 0.001]. The direct effects of stress and gender on school bullying were statistically significant, as was the effect of stress*gender interaction on school bullying (coeff = −0.241, *t* = −7.68, 95% CI = −0.303 ~ −0.180). Details are shown in Table 3.

**Table 3:** Effects of stress and gender on bullying behavior in school.

	*R* ^2^	coeff	se	*t*	*p*	LLCI	ULCI
Stress (*X'*)	0.126^***^	0.645	0.051	12.7	0.000	0.545	0.744
Gender (*M'*)		−0.370	0.032	−11.679	0.000	−0.432	−0.307
Stress*Gender (*X'M'*)		−0.241	0.031	−7.680	0.000	−0.3030	−0.180

^*^p < 0.05, ^**^p < 0.01, ^***^p < 0.001.

**Figure 2:**
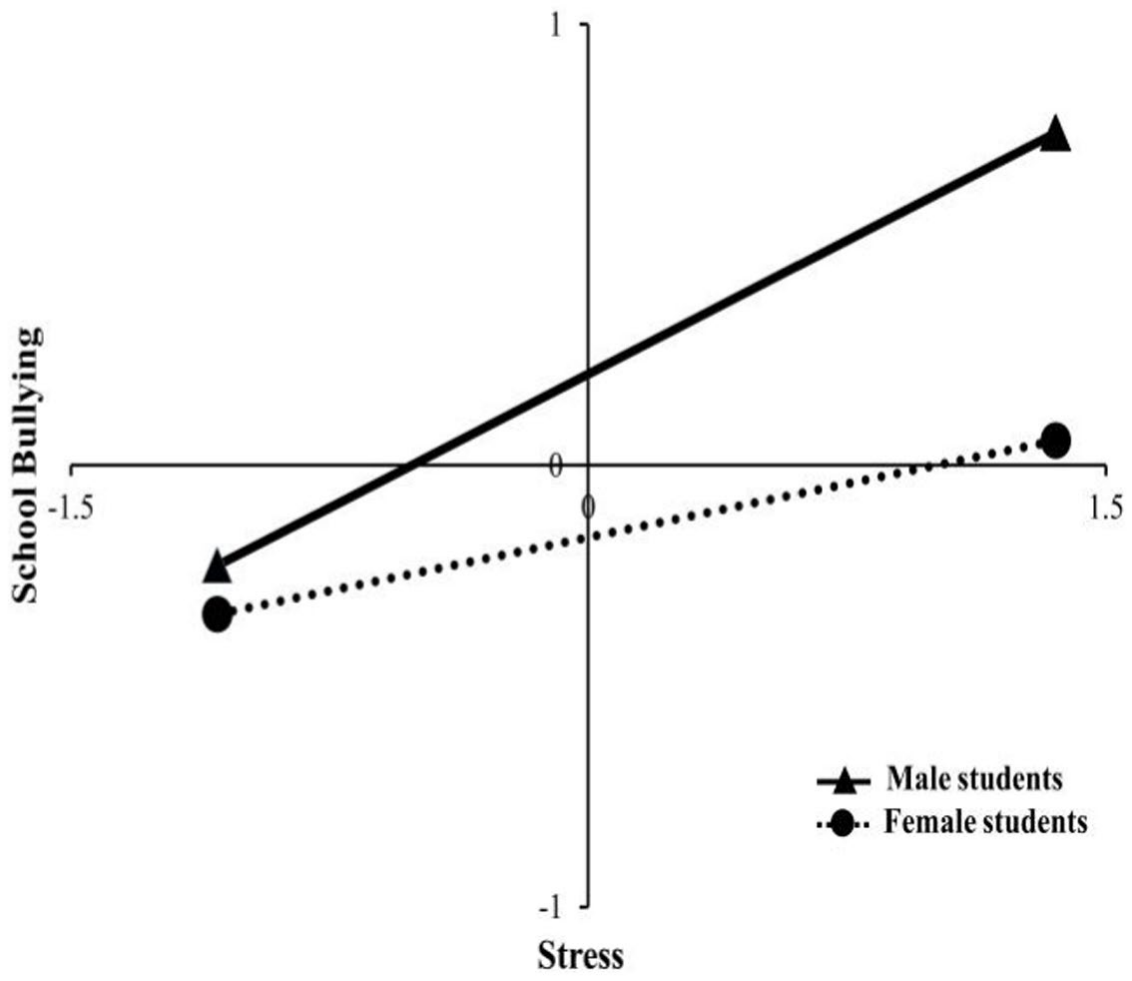
Moderating role of gender in the relationship between stress and school bullying behavior.

Direct and moderating effect analyses were conducted to further test the effect of stress on school bullying behavior under gender-specific moderation. The analysis found a statistically significant predictive effect of stress on school bullying in both genders. In the male group, the effect values for the effect of stress on school bullying were larger (Effect = 0.404, *t* = 17.525, 95% CI = 0.358 ~ 0.449) than those in the female group (Effect = 0.162, *t* = 7.580, 95% CI = 0.120 ~ 0.204). A slope analysis revealed (Figure 2) that school bullying intensified with the increasing stress in both genders, however, the effect of stress on school bullying was higher in the male group than in the female group. It is evident that gender moderates the effect of stress on school bullying.

#### 3.2.2. The moderating role of grade level in stress and school bullying behavior

As shown in Table 4, the coefficients of the established model and the interaction effects are statistically significant; furthermore, the model meets the fit criteria. The Bootstrap moderating effect analysis found that stress and grade level significantly predicted school bullying and explained 12.47% of the total variance in school bullying behavior [*F*(3, 3,562) = 169.181, *p* < 0.001]. The interaction between the first two could explain 1.35% of the total variation [*F*(1, 3,562) = 54.95, *p* < 0.001]. The direct effects of stress and grade level on school bullying were statistically significant (coeff = −0.628, *t* = 12.610, 95% CI = −0.530 ~ −0.725; coeff = −0.366, *t* = −11.66, 95% CI = −0.428 ~ −0.305), as was the effect of stress*grade interaction on school bullying (coeff = −0.232, *t* = −7.413, 95% CI = −0.294 ~ −0.171).

**Table 4:** Effects of stress and grade level on school bullying.

	*R* ^2^	coeff	se	*t*	*p*	LLCI	ULCI
Stress (*X'*)	*0.125* ^***^	0.628	0.050.	12.610	0.000	0.530	0.725
Grade (*M'*)		−0.366	0.031	−11.660	0.000	−0.428	−0.305
Stress*Grade (*X'M'*)		−0.232	0.031	−7.413	0.000	−0.294	−0.171

^*^p < 0.05, ^**^p < 0.01, ^***^p < 0.001.

**Figure 3:**
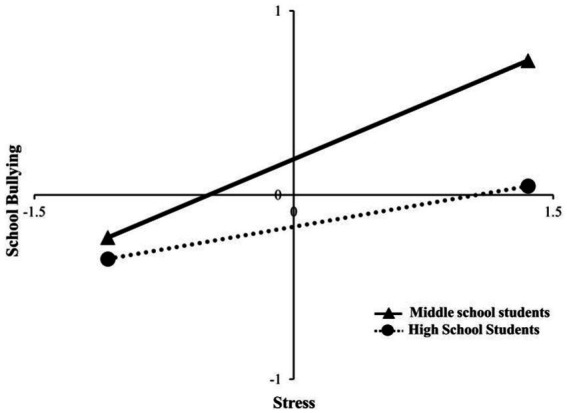
Moderating role of grade level in the relationship between stress and school bullying behavior.

Direct and moderating effect analyses were conducted to further examine the effects of stress on school bullying behavior under grade-specific moderation. The analysis found a statistically significant predictive effect of stress on school bullying at both grade levels. In the middle school group, the effect values of stress on school bullying were larger (Effect = 0.395, *t* = 17.708, 95% CI = 0.352 ~ 0.439). In the high school group, the effect value of stress on school bullying was smaller (Effect = 0.163, *t* = 7.340, 95% CI = 0.120 ~ 0.206). A slope analysis revealed (Figure 3) that the school bullying behavior intensified with increasing stress, in both groups. However, the effect of stress on school bullying behavior was higher in the middle school group than in the high school group, showing that grade level moderates the effect of stress on school bullying.

## 4. Discussion

### 4.1. The relationship between stress and school bullying

This study explored the relationship between stress and school bullying behaviors among secondary school students as well as its mechanisms of action. Results found that stress had a significant positive predictive effect on school bullying behavior, which is consistent with previous national and international studies (Estrada-Martiner et al., 2012; Herts et al., 2012; Hsieh et al., 2014; Wang and Zhang, 2015; Shao et al., 2018; Hu et al., 2021), and the higher the stress level of an individual, the higher the risk of developing school bullying behavior. The “frustration–aggression” theory suggests that when their needs are not met, individuals may feel frustration and use aggression to relieve and mask it. When students experience certain stressful events in their daily lives, they may feel frustrated in achieving their goals or even feel that their interests or safety are threatened, which can provoke a differential response, thus gradually developing reactive aggression to defend and protect themselves. Secondary school students in the adolescent stage have not yet completed/integrated the appropriate cognitive-emotional mechanisms to properly handle various stressful situations. They are characterized by immature mental development, mood swings, and lack of self-control, therefore, reactive aggression caused by frustration is more likely to occur. Consequently, to mitigate the effects of stress on school bullying behaviors of secondary school students, families and schools should actively focus on those facing stressful events at school and help them channel their emotions in a timely manner as well as to properly navigate through the crisis.

### 4.2. The moderating role of gender in the relationship between stress and school bullying behavior

This study found that the effect of stress on school bullying behavior of secondary school students was moderated by gender, with male students being more likely to develop school bullying behavior after being affected by stress. Buss proposed the theory of “gender differences in aggression” (Buss et al., 1998), which suggests that men and women differ in the targets, behaviors, and causes of aggression. Anthropological, physiological, and evolutionary studies have revealed that genetic factors explain 50% of the variance in aggressive behavior (Miles and Carey, 1997), with gender playing a moderating role between genes and aggression, and that genetic polymorphisms are significantly associated with the occurrence of aggressive behavior mainly in male populations (Lachman et al., 1998; Estrada-Martiner et al., 2012). Moreover, there are large gender-based differences in stress responses (Atkinson and Waddell, 1997), and a study by Bernhard et al. (2021) revealed that alterations in sex hormones (increased androgens in males and decreased estrogens in females) are strongly associated with conduct disorder (Bernhard et al., 2021), with the male group being more affected, in multiple ways, than the female group. In addition, the socialization theory of gender roles mentions that men and women show gender differences in many aspects due to the different roles and responsibilities they hold in the socialization process (Beall and Sternberg, 1995). The gender role stereotype that males should be assertive, independent, competitive, and aggressive, while females should be affectionate, caring, sensitive to the demands of others, and cooperative, lead to different gender characteristics and behaviors for men and women, making the former more likely to be involved in school bullying than the latter. Furthermore, both genders differ in the resolution strategies they use when faced with a problem that needs to be solved (Chen, 2011), with girls being stronger in “seeking social support” and problem-oriented coping strategies (Frydenberg and Lewis, 2000; Eschenbeck et al., 2007), while boys tend to use the strategy of “distracting themselves from unpleasant emotions” by distancing themselves more from the problem and becoming less emotionally invested in it. This increases their susceptibility to externalizing behaviors. As girls mature earlier and engage in more socialization than boys, they use more problem-solving strategies when faced with stress, generally employing non-aggressive approaches to conform their behavior to socially accepted role requirements; thus, females are less likely to use aggression as a form of problem solving than males. Consequently, schools should establish precise school bullying prevention and control mechanisms to give adequate attention to male high school students in stressful situations.

### 4.3. The moderating role of grade level in the relationship between stress and school bullying

This study found that stress has an impact on school bullying behaviors among middle school students while being moderated by grade level, with middle school students more likely to be affected by stress and engage in school bullying than high school students. Previous research has suggested that aggression and victimization are highly stable from late childhood to adolescence (Scholte et al., 2007); however, research findings of Ryoo et al. (2015) showed that bullying and victimization are dynamic phenomena in groups that are frequently involved in bullying, suggesting that interventions should address the heterogeneity of the experiences of those involved in school bullying during adolescence. The present study further confirms this idea. The development of adolescents’ coping styles depends on the cognitive, biological, emotional, and social development state (Backhaus et al., 2010), and research has confirmed that coping strategies diverge and expand with age, showing some flexibility (Zimmer-Gembeck and Skinner, 2011), especially in the cognitive domain (Beck et al., 2016). According to the Social Information Processing Model of Children’s Social Adaptation (SIP model) proposed by Crick and Dodge (1994) children have a specific social cognitive process that influences or determines the final behavioral response before they respond to a particular social situation in the face of stress. High school students with higher cognitive levels are more inclined to make more comprehensive and objective interpretations at all stages of the information processing process, thus enabling appropriate behavioral responses. Zhang et al. (2011) further confirmed that dialectical thinking can reduce the tendency for aggressive behavior. Moreover, as secondary school students advance in their cognitive level, they also significantly develop their social emotions, increasing their ability to reflect on their emotions and developing an increasingly sophisticated emotion regulation strategy that manifests as “positive self-affirmation” (Zimmer-Gembeck and Skinner, 2011). As high school students get older and socialize more, they are better able to discipline themselves with social norms and control their own behavior, they begin to seek the approval and support of others, they naturally behave in a way that is consistent with social morality, and social moral norms are gradually internalized. Chen’s study concluded that children become more socialized and use more problem-solving strategies as they get older, more so after the age of 12 (Eschenbeck et al., 2007). Therefore, high school students at an older age will be less likely to engage in school bullying than middle school students, and more attention should be paid to the prevention and control of school bullying on middle school campuses and the establishment of a comprehensive prevention and control system.

This study has some limitations. First, the sample size was small and limited to secondary school students in Guizhou Province. Therefore, a larger and broader sample is needed to confirm our findings in future studies. Second, this study relied on secondary school students’ self-reports to conduct the assessment. Although the scale used in this study had high reliability, it could be further confirmed in the future using more rigorous and objective methods. Third, we did not adequately consider the effects of other potentially relevant variables (e.g., confounding factors such as coping strategies) when examining the relationship between stress and school bullying, which should be considered in future research.

## 5. Conclusion

The present study preliminarily revealed that stress has a significant effect on school bullying behavior among secondary school students, with gender and grade level playing a moderating role between stress and school bullying behavior. In terms of the effect of stress on school bullying behavior, males were more likely to be affected than female and Junior high school students than high school students. This may guide school bullying intervention programs on secondary school campuses. For example, society should pay attention to the bullying behavior of boys and focus on the problem of bullying in junior high school.

## Data availability statement

The raw data supporting the conclusions of this article will be made available by the authors, without undue reservation.

## Ethics statement

The studies involving human participants were reviewed and approved by School of Educational Sciences, Anshun University, China. Written informed consent to participate in this study was provided by the participants’ legal guardian/next of kin.

## Author contributions

SL was responsible for study design, data analysis and interpretation, and manuscript completion. YB was responsible for the analysis, interpretation of data, and manuscript completion. TQ and CL were responsible for data collection. All authors contributed to the article and approved the submitted version.

## Conflict of interest

The authors declare that the research was conducted in the absence of any commercial or financial relationships that could be construed as a potential conflict of interest.

## Publisher’s note

All claims expressed in this article are solely those of the authors and do not necessarily represent those of their affiliated organizations, or those of the publisher, the editors and the reviewers. Any product that may be evaluated in this article, or claim that may be made by its manufacturer, is not guaranteed or endorsed by the publisher.
